# Vagal neuropathies: An underrecognized potential driver of physical, mental, and immune symptoms

**DOI:** 10.1016/j.bbih.2026.101252

**Published:** 2026-05-11

**Authors:** Bandy Chen, Laurent Gautron

**Affiliations:** a UC San Diego, School of Medicine, La Jolla, CA, 92093, USA; b Department of Internal Medicine and Center for Hypothalamic Research, UT Southwestern Medical Center, 5323 Harry Hines Blvd, Dallas, TX, 75390, USA

## Abstract

Peripheral neuropathies are widely recognized disorders of the peripheral nervous system. However, research and clinical attention have focused predominantly on somatosensory, somatic motor, and sympathetic neurons, with comparatively little emphasis on the vagus nerve. In this review, we propose a working definition of vagal neuropathies, referring to pathological alterations affecting vagal sensory and/or motor neurons. We synthesize evidence for vagal neuropathy across diverse human diseases and experimental models, examining structural, functional, and molecular hallmarks of nerve injury, associated clinical manifestations, and diagnostic challenges. Emphasis is placed on immune–neural interactions, given the central role of the vagus nerve in inflammatory reflexes and immunomodulation, and because immune and inflammatory mechanisms are implicated in the pathogenesis of many other neuropathies. Collectively, the literature suggests that vagal neuropathy is more prevalent than currently appreciated and may contribute to a broad spectrum of physical, mental, and immune dysfunctions. Recognizing vagal neuropathy as a distinct and clinically relevant entity has important implications for diagnosis, mechanistic understanding, and therapeutic development.

## 1 The vagus nerve in health, disease, and immunity

The vagus nerve is a mixed cranial nerve composed predominantly of sensory (afferent) fibers (Prescott and Liberles, 2022), with cell bodies in the nodose and jugular ganglia that convey interoceptive information from thoracic and abdominal organs, including the heart, aorta, lungs, airways, pancreas, and nearly the entire gastrointestinal tract (Spencer et al., 2025; Ottaviani and Macefield, 2022). Motor (parasympathetic) efferent fibers arise primarily from neurons located in the dorsal motor nucleus of the vagus (DMV) and the nucleus ambiguus (Wang et al., 2025). These neurons are cholinergic and innervate cardiac tissue, airway, and postganglionic neurons of the enteric nervous system (Wang et al., 2025). The vagus also provides sensory and motor innervation to the vocal cords via its laryngeal branches (Suzuki et al., 2020). Although certain tissues such as adipose tissue and the spleen lack direct vagal innervation, they are influenced indirectly through vagal–sympathetic interactions and immune-mediated pathways (Huston et al., 2009; Berthoud et al., 2025). Advances in molecular profiling, including single-cell RNA sequencing, have fundamentally revised the classical view of the vagus nerve as a uniform structure (Zhao et al., 2022). Instead, it is now understood to comprise molecularly, anatomically, and functionally distinct subpopulations of sensory and motor neurons, each with specific targets, signaling modalities, and immune sensitivities (Egerod et al., 2019; Steinberg et al., 2016).

Functionally, the vagus nerve mediates numerous essential reflexes, including coughing, vomiting, baroreflexes, respiratory reflexes, and gastric accommodation through which it regulates breathing, digestion, cardiovascular function, and energy balance (Berthoud et al., 2021; Sclafani and Kramer, 1985; Dalli et al., 2017). Beyond homeostatic regulation, vagal afferent signaling contributes to higher-order processes such as cognition, mood, and behavior (Hays et al., 2013). Critically, the vagus nerve has also emerged as a regulator of immunity. Detailed descriptions of the vagal regulation of immunity have been extensively reviewed elsewhere (Andersson and Tracey, 2011; Chavan and Tracey, 2017; Matteoli and Boeckxstaens, 2013). Instead, we emphasize several essential features relevant to this review. Through the so-called inflammatory reflex, vagal afferents detect peripheral inflammatory signals, while vagal efferents suppress excessive cytokine production via cholinergic mechanisms (Tracey, 2002). This neuro–immune circuitry modulates innate and adaptive immune responses in organs such as the gut, lung, and spleen, and plays a role in host defense, immune tolerance, and resolution of inflammation.

Given these extensive anatomical connections and functional roles, one might expect vagal pathology to feature prominently in modern medicine. While vagal neuropathy is a clinically recognized entity (e.g. laryngopharyngeal and cardiovagal neuropathies) (Benninger and Campagnolo, 2018; Muppidi et al., 2011), however, most research has focused on the role of vagal neurons in physiological regulation rather than their involvement in disease etiology. Few human disorders have been explicitly framed as associated with vagal neuropathy—that is, disease or damage of the vagus nerve itself. Despite the broad physiological roles of the vagus nerve, disorders affecting this nerve remain comparatively under-studied and incompletely characterized. Vagal neuropathies can potentially produce a wide spectrum of symptoms involving many organs across multiple clinical specialties. As a result, their diagnosis and pathophysiology remain not fully mechanistically understood, and the literature addressing these conditions is dispersed across several fields. At the same time, increasing evidence points to immune and inflammatory mechanisms contributing to peripheral neuropathies, raising the possibility that similar processes may underlie some vagal nerve disorders. These considerations provide a strong rationale for examining vagal neuropathies and synthesizing current knowledge regarding their mechanisms, clinical manifestations, and potential therapeutic implications.

## 2 Vagal neuropathies as clinical entity

### 2.1 Definition

Neuropathy is broadly defined as damage to or disease of peripheral nerves (Mauermann and Staff, 2025; Frisaldi, 2025) (see also Box 1). The term encompasses a heterogeneous group of disorders that vary according to etiology, neuronal subtype, anatomical distribution, and clinical presentation (Mauermann and Staff, 2025; Frisaldi, 2025). Common classifications include polyneuropathy, autonomic neuropathy, inherited neuropathy, toxic neuropathy, and idiopathic neuropathy. Major causes of peripheral neuropathy—including diabetes, chronic alcohol use, infections, chemotherapeutic agents, autoimmune diseases, genetic disorders, surgical injury, and aging (Mauermann and Staff, 2025). Peripheral neuropathy affects approximately 2% of the general adult population, with prevalence rising to nearly 7% among individuals over 60 years of age (Hoffman et al., 2015). These figures likely underestimate the true burden of disease, as early-stage or mild neuropathies often go undiagnosed.

Box 1 **Operational criteria for defining vagal neuropathy**

A central conceptual issue in this review is the distinction between neural plasticity and neuropathy. This distinction is inherently complex: processes interpreted by some authors as plasticity may reasonably be considered neuropathic by others. Rather than attempting to resolve this broader debate, we outline here the operational framework used throughout this manuscript and indicate where neuropathy is well established versus where it remains difficult to distinguish from neuroplastic changes.

Historically, plasticity referred primarily to adaptive changes in the nervous system associated with beneficial outcomes such as learning or recovery. Over time, the concept has broadened and is now often applied to neural responses occurring in disease. While this expanded usage recognizes that neural responses may be adaptive or maladaptive, it can also blur conceptual boundaries when most neural alterations are described as plasticity.

For clarity, we use plasticity primarily to refer to non-pathological processes such as development, physiological adaptation, or reversible functional modulation. In contrast, neuropathy in its medical sense denotes a diseased state of the peripheral nervous system, typically involving structural or functional alterations associated with pathology and symptoms. In this review, we adopt a broad operational definition of neuropathy as neural changes occurring in association with disease, regardless of whether they are definitively maladaptive or directly causal of symptoms.

Operational indicators suggesting vagal neuropathy in this review include:

1. Neural changes occurring in the context of established disease states
2. Persistent alterations in neuronal excitability or signaling beyond normal physiological adaptation
3. Presence of molecular or cellular markers commonly associated with neuronal stress or injury (e.g., ATF3 expression)
4. Structural, transcriptional, or neurochemical alterations resembling those described in other types of peripheral neuropathies
5. Chronic or progressive cellular changes that are unlikely to represent transient physiological adaptation

In this manuscript, we therefore preferentially use the term neuropathy when such features are present, while acknowledging that early or transitional states may remain difficult to distinguish from neuroplastic responses.

In early disease stages, distinguishing plasticity from neuropathy may be difficult, and these states may represent transitional or pre-neuropathic processes. Indeed, many alterations described in vagal neurons—including changes in excitability, cellular stress responses, and neuropeptide expression—resemble features widely recognized as neuropathic in other peripheral neurons (e.g., dorsal root ganglia). At the same time, demonstrating clear structural injury in the vagus nerve remains challenging due to the limited available literature, and we highlight throughout the manuscript where additional evidence is needed.

Conceptually, neuropathy and plasticity are therefore not mutually exclusive. Plasticity may represent a potentially reversible, pre-neuropathic state, whereas established neuropathy may be more persistent without necessarily representing a fundamentally different biological process.

Alt-text: Box 1

**Fig. 1:**
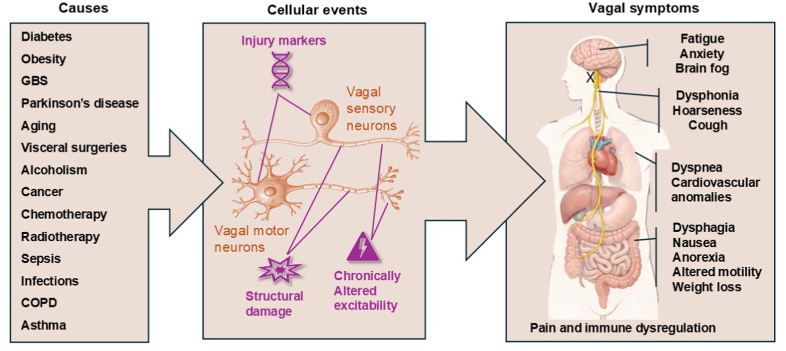
**Overview of vagal neuropathies as a clinical entity.** This schematic summarizes the most common suspected causes, anatomical sites, and potential symptoms associated with vagal neuropathies. The etiologies of vagal neuropathies largely overlap with those of painful peripheral neuropathies affecting the extremities and include, among others, cancer, diabetes, infection, and immune-mediated mechanisms. The pathophysiology of vagal neuropathy depends on the underlying cause and the neuronal populations involved but generally includes some degree of nerve injury, altered electrophysiological excitability, and phenotypic changes characterized by the induction of neuronal injury markers. Vagal neuropathy is most likely to occur in the airways, lungs, and gastrointestinal tract, which are heavily innervated by the vagus nerve, contain large populations of immune cells, and are frequent sites of infection and inflammation. Clinical manifestations of vagal neuropathy and dysfunction encompass a broad range of respiratory and gastrointestinal symptoms, which often prove refractory to treatment and may persist over long periods. Because the vagus nerve conveys sensory information to the brain, associated symptoms may also include fatigue, anxiety, and cognitive dysfunction (“brain fog”). In addition, given its role in pain modulation and immune regulation, vagal neuropathy may be linked to chronic pain and dysregulated immunity or recurrent infections. The images of the neurons and human body outline were generated using ChatGPT prompts and the labels were added in Microsoft Power Point.

By analogy, vagal neuropathies should be conceptualized as a heterogeneous spectrum of conditions affecting vagal sensory and/or motor neurons. Moving forward, vagal neuropathies should be categorized according to the type of neurons affected (sensory vs motor), underlying cause (e.g., metabolic, immune-mediated, toxic, genetic), and target organs or reflex circuits involved (e.g., gastric vagal neuropathy, cardiovascular vagal neuropathy) (Fig. 1).

Whereas vagal neuropathy is likely to coexist with other types of neuropathies, the prevalence of vagal neuropathy remains unknown. This gap reflects the absence of a coherent conceptual framework, the heterogeneity of vagal neuron subtypes, and the lack of standardized diagnostic tools. This review aims to serve as a foundation for future efforts in this area. We do not propose a redefinition of vagal neuropathy but rather aim to integrate existing clinical frameworks with emerging mechanistic and systems-level perspectives.

### 2.2 Pathophysiology

The pathophysiological mechanisms leading to neuropathy are diverse and context dependent. In diabetes, chronic hyperglycemia and metabolic stress predominate; in toxic neuropathies, abnormal DNA binding or mitochondrial dysfunction may be central; in immune-mediated neuropathies, excessive inflammation and autoantibody-mediated injury are key drivers. Despite this heterogeneity, several shared features characterize many neuropathies.

#### 2.2.1 Structural damage

Structural neuronal injury is a defining feature of most neuropathies (Fig. 1). Axonopathy, typically involving degeneration of distal nerve terminals, is common (Stino and Smith, 2017). Demyelination represents another major mechanism and is characteristic of disorders such as Guillain–Barré syndrome (GBS) (Kieseier et al., 2018). In some conditions, neuronal death at the level of the cell body occurs, although the extent of cell loss varies substantially by etiology (Swett et al., 1995).

In animal models, direct evidence of vagal nerve injury can be obtained using electron microscopy, neurotropic tracers, and genetically encoded reporters. These approaches reveal axonal degeneration, altered terminal morphology, demyelination, and loss of neurons. Vagal sensory neurons appear capable of limited regeneration following injury, whereas efferent neurons may be less resilient, suggesting differential vulnerability and recovery potential (Phillips et al., 2003).

In humans, direct histological assessment of the vagus nerve is rarely feasible and is generally restricted to postmortem studies or cases involving surgical resection (Havton et al., 2021). Noninvasive imaging modalities such as ultrasonography can detect gross abnormalities, including nerve enlargement, but substantial interindividual variability limits diagnostic sensitivity. CT, MRI, and PET imaging may identify secondary causes of injury—such as tumors or inflammation—but do not directly assess axonal integrity (Rangavajla et al., 2014).

#### 2.2.2 Chronically altered excitability and phenotypic switching

Altered neuronal excitability is a hallmark of many neuropathies and often underlies clinical symptoms (Fig. 1). Pharmacological evidence supports this link, as local anesthetics can alleviate neuropathic pain by reducing aberrant neuronal firing (Raja et al., 2020). However, altered excitability does not necessarily imply neuropathy; reversible neuroplasticity may occur in response to transient inflammation or metabolic stress (Prescott et al., 2014) (Box 1). In contrast, neuropathy-related changes are typically persistent and accompanied by structural and/or molecular injury markers (Gold and Gebhart, 2010).

At the molecular level, peripheral nerve injury induces characteristic transcriptional changes, including downregulation of neuron-specific genes and upregulation of stress, survival, and regeneration-associated pathways (Renthal et al., 2020). Altered neuropeptide and neuropeptide receptor expression is also common in injured peripheral neurons (Regalia et al., 2002). In experimental models, activating transcription factor 3 (ATF3) is widely used as a marker of neuronal injury and is robustly induced in vagal neurons following axotomy, inflammation, or toxic exposure (Seijffers et al., 2007).

Whether this pattern holds true in humans is less clear, as molecular changes in animal models are typically examined shortly after injury, whereas human data are often derived from postmortem tissue at later disease stages (Sapio et al., 2016; Hall et al., 2022). Moreover, in humans, direct electrophysiological recordings from the vagus nerve and molecular profiling of vagal neurons are rarely feasible. Clinicians rely instead on indirect measures such as heart rate variability and baroreflex sensitivity to infer vagal function. While useful, these surrogate markers lack specificity and cannot distinguish vagal neuropathy from other autonomic influences (Burger et al., 2020).

### 2.3 Diagnosis

The diagnosis of peripheral neuropathy in human patients begins with a thorough assessment of symptoms, such as sensory loss, pain, and weakness, as well as clinical signs such as reduced reflexes or altered sensation (Watson and Dyck, 2015). Nerve conduction studies and electromyography are essential for confirming the presence of peripheral neuropathy and distinguishing between axonal and demyelinating forms. Additional investigations, including blood tests (e.g., glucose levels, vitamin deficiencies, autoimmune or infectious markers), genetic testing, and skin biopsy to measure intraepidermal nerve fiber density, can help identify underlying causes. Unfortunately, the latter tests are not applicable to the human vagus nerve.

Vagal neuropathy may give rise to a broad range of autonomic, sensory, and metabolic symptoms, depending on the neuronal subtypes involved and the extent and chronicity of injury (Fig. 1). In clinical practice, identifying vagal neuropathy is particularly challenging, as diagnosis is usually inferred from nonspecific or overlapping symptoms rather than direct evidence of nerve damage. More clearly recognized manifestations of vagal involvement include voice hoarseness (Yamamoto et al., 2025), vocal cord paralysis (Holinger et al., 1976), dysphagia (Miles et al., 2022), chronic cough (Al-Biltagi et al., 2022; Wu et al., 2023), dyspnea (Nishino, 2009), and baroreflex failure (Lamotte et al., 2021).

Other commonly reported symptoms that may involve disrupted vagal signaling or injury—but are considerably more difficult to attribute with certainty—include fatigue (VanElzakker, 2013), cognitive complaints or “brain fog” (Wong et al., 2023), anxiety (Pena et al., 2013), and anorexia (Russek, 1971). The mechanistic link between vagus nerve integrity and mental health remains elusive. However, studies on vagus nerve stimulation and vagal deafferentation suggest a functional connection between the vagus nerve and cognition, memory, and mental health (Engineer et al., 2017; Klarer et al., 2017).

With respect to pain, vagal afferents influence nociception primarily through indirect modulation of descending pain control pathways (Randich and Gebhart, 1992). Consequently, vagal dysfunction may exacerbate pain perception, not necessarily by generating pain directly, but by altering central pain modulation or interacting with neuropathies in other peripheral nerves. Overall, establishing a diagnosis of vagal neuropathy in humans remains difficult. Clinical clues supporting a neuropathic origin include symptom persistence over time, refractoriness to anti-inflammatory treatments, partial responsiveness to neuropathic pain medications (e.g., gabapentin), and convergence with other criteria such as evidence of structural damage or sustained functional impairment.

### 2.4 Treatments

When possible, addressing the underlying etiology should be the primary goal of treatment. For example, glycemic control is crucial in the management of diabetic neuropathy. However, in some cases, the root cause cannot be identified, or no known cure exists. This is often the case for inherited or trauma-related neuropathies. In these instances, symptom management becomes the focus of treatment. Patients with neuropathic pain may benefit from various pharmacological options, including gabapentinoids, serotonin-norepinephrine reuptake inhibitors, tricyclic antidepressants, and topical agents (D'Souza et al., 2022). Other treatment approaches may include nerve decompression surgery, electrical neuromodulation, and cognitive therapy (i.e. cognitive‐behavioral techniques applied to chronic pain perceptions), among others (Zeldin et al., 2025; Gibson et al., 2017).

Therapeutic strategies directly targeting the vagus nerve remain limited. While pharmacological cholinergic agents can modulate vagal efferent outflow (Kakinuma et al., 2009), there are no drugs specifically targeting vagal afferents pathways. An emerging exception is the electrical neuromodulation of the vagus nerve. Vagus nerve stimulation (VNS) has been approved for treatment-resistant epilepsy and depression (Shi et al., 2025). Nevertheless, the overall number of individuals currently receiving vagus-targeted therapies remains relatively small; only 130,000 patients have been implanted with vagal stimulators worldwide since the late 1990's (Zanello et al., 2025). In other words, the vagus nerve occupies a surprisingly small niche in modern medicine for a structure connected to numerous vital organs and functions. Admittedly speculative, it is nonetheless tempting to propose that VNS could be used as a tool to modulate vagally mediated inflammatory and physiological processes perturbed during neuropathy.

### 2.5 Why has vagal neuropathy been neglected?

Several factors contribute to the neglect of vagal neuropathy. First, vagal sensory dysfunction rarely produces overt pain, leading to diffuse and nonspecific symptomatology. As noted by Dr. Cervero (1994), “… the apparent insensitivity of viscera to noxious stimuli led many investigators to believe that internal organs lacked afferent nerves.” Consequently, symptoms arising from vagal sensory neuropathy would be expected to be diffuse, nonspecific, and difficult to attribute to a discrete biological reason. Manifestations such as dysphagia, altered heart rate, or blood pressure dysregulation may be easily attributed to stress or secondary effects of illness and its treatments rather than to an underlying vagal nerve pathology.

Second, diagnostic access to vagal fibers is limited, and validated biomarkers are lacking. While skin biopsy and electromyography are feasible and widely used to assess neuropathy in extremities, access to vagally innervated tissues is far more limited. As a result, the diagnosis of vagal neuropathy is complex and often relies on indirect or surrogate markers rather than direct structural or molecular evidence of nerve injury. Because biopsies of visceral organs are uncommon in humans, reliable markers of vagal fibers are lacking.

Third, neuropathy research has historically emphasized painful conditions, shaping both experimental and clinical perspectives. As a result, vagal dysfunction is often framed as neuroplasticity rather than neuropathy, even when changes are persistent and injury-associated.

## 3 Vagal neuropathies as a unifying feature across diseases

Across metabolic, infectious, inflammatory, autoimmune, neurodegenerative, surgical, and toxic conditions, converging evidence indicates that vagal neurons undergo structural, functional, and molecular alterations consistent with neuropathy or pre-neuropathic states. Immune-mediated mechanisms—cytokine exposure, immune cell infiltration, demyelination, and sustained neuro–immune signaling—recur as central drivers.

### 3.1 Diabetes and obesity

A substantial body of literature documents vagal nerve damage in diabetes. Electron microscopy studies provide direct structural evidence of vagal neuropathy, demonstrating axonal loss through fiber counts in resected vagal nerves from patients with both type 1 and type 2 diabetes (Guy et al., 1984; Britland et al., 1990; Guo et al., 1987), as well as pronounced ultrastructural axonal abnormalities in diabetic rodents (Yagihashi and Sima, 1986). Histological analyses in animal models further corroborate these findings, revealing atrophic terminal endings of vagal fibers innervating the cardiovascular system (Li et al., 2010) and loss of vagal neurons (Lee et al., 2002).

Functional alterations of vagal neurons in diabetes are also well documented. Electrophysiological recordings from the nodose ganglion in STZ-treated rats demonstrate significant changes in neuronal excitability and firing properties (Tu et al., 2010; Li et al., 2008; da Silva-Alves et al., 2023). Notably, distinct subpopulations of vagal afferents respond differently to the diabetic milieu, indicating potential cell-type-specific vulnerability. These functional changes are accompanied by alterations in vagal neurochemistry and neuropeptide expression (Regalia et al., 2002), consistent with an injury-associated or maladaptive phenotype.

Clinically, diabetes frequently manifests with autonomic symptoms, particularly involving cardiovascular and gastrointestinal systems. Common features include syncope, impaired gastric acid secretion, dysmotility, and altered blood pressure regulation. Numerous clinical studies report such abnormalities in patients with both type 1 and type 2 diabetes (Kornum et al., 2024; Wegeberg et al., 2021; Undeland et al., 1997; Ziegler et al., 2018). Collectively, these findings convincingly indicate that poorly controlled diabetes is likely to result in some degree of vagal neuropathy, typically coexisting with neuropathy of other peripheral nerves. The proportion of diabetic patients affected remains unclear; however, if vagal involvement mirrors the prevalence of somatosensory neuropathy, a substantial fraction of patients may be affected.

Type 2 diabetes is strongly associated with obesity, raising the question of whether obesity alone, independent of hyperglycemia, is sufficient to induce vagal neuropathy. Epidemiological studies indicate that obese individuals without diabetes frequently exhibit peripheral neuropathy, albeit at lower prevalence than diabetic patients (Callaghan et al., 2016; Bonomo et al., 2020; Tierney et al., 2022).

Electrophysiological studies report hypo-responsiveness of vagal afferent neurons to mechanical deformation and metabolic signals in high-calorie–fed rodents (Troy et al., 2016; Park et al., 2018; Loper et al., 2021; Kentish et al., 2018; Wang et al., 2019). Additional findings include altered firing of DMV neurons (Clyburn et al., 2018) and vagal motor dysfunction affecting cardiovascular control (Strain et al., 2023). Despite these observations, it remains unclear whether obesity alone induces true vagal neuropathy or instead reflects neuroplasticity in response to chronic overnutrition. Importantly, convincing histological or molecular evidence of vagal axonal degeneration in obese, non-diabetic animals is currently lacking (Yuan et al., 2016). Moreover, many experimental models fail to clearly dissociate obesity from diabetes, complicating interpretation.

Paradoxically, rapid weight loss following bariatric surgery represents a double-edged sword: while it may ameliorate diabetes-associated neuropathy, it also increases the risk of nutritional deficiencies, a well-established cause of peripheral neuropathy (Aghili et al., 2019).

### 3.2 Infections, sepsis, and endotoxemia

Severe viral infections, such as influenza, may be followed by painful peripheral neuropathy (Kaida et al., 1997), although autonomic involvement is reported less frequently. The mechanisms underlying infection-induced neuropathy are likely multifactorial and incompletely understood, with excessive inflammation, cytokine-mediated injury, and immune cell infiltration representing plausible contributors (Verzele et al., 2024). In addition, postmortem analyses of the brainstem and vagus nerve from patients who died of COVID-19 reveal SARS-CoV-2 RNA within vagal nerve samples (Woo et al., 2023). Similarly, influenza virus can directly infect vagal sensory neurons in mice (Matsuda et al., 2004). Together, these findings support the hypothesis that certain viral pathogens may directly “infect” vagal neurons.

However, direct structural evidence for infection-related vagal neuropathy remains limited. In patients with post–COVID-19 syndrome, ultrasonographic enlargement of the vagus nerve has been reported, suggesting inflammatory or structural alterations (Llados et al., 2024). In rodents, acute immune challenges increase vagal excitability (Diniz et al., 2018) and such changes persist chronically beyond infection for at least 6 weeks (Strohl et al., 2025). At the molecular level, induction of ATF3 in vagal sensory neurons following LPS exposure or influenza infection indicates substantial neuronal stress and raises the possibility of axonal injury (Kaelberer et al., 2020; Almanzar et al., 2025). Moreover, Sendai virus infection induces transient tachykinin expression in vagal afferents, which resolves within weeks (Carr et al., 2002; Wester et al., 1991). Upregulation of tachykinins in sensory neurons is a phenomenon linked to neuropathic states (Marchand et al., 1994).

Survivors of severe infection or sepsis commonly experience persistent, nonspecific symptoms—including fatigue, reduced well-being, and increased susceptibility to recurrent infections—that may persist for months or years (Choutka et al., 2022). Persistent gastroparesis following viral infection has been described clinically, albeit rarely (Bityutskiy et al., 1997). In COVID-19, symptoms consistent with vagal involvement—including vocal cord paralysis and dysphagia—have been reported (Rapoport et al., 2022). Collectively, these observations raise the possibility that vagal neuropathy contributes to post-infectious syndromes, particularly given the overlap with symptoms of autonomic dysfunction, chronic cough, weight loss, dysphagia, persistent inflammation, and fatigue. Nonetheless, given the paucity of structural and electrophysiological data, vagal neuropathy cannot yet be diagnosed with high confidence in most post-infectious contexts.

### 3.3 Non-infectious inflammatory diseases

Non-infectious inflammatory insults to the gastrointestinal tract, such as inflammatory bowel disease, colitis, and peptic ulcers, would be expected to impair vagal integrity. In rodent models of dextran sodium sulfate–induced colitis or acetic acid–induced gastric ulcers, nodose ganglion neurons exhibit altered calcium signaling and electrophysiological hyperexcitability (Huerta et al., 2025; Dang et al., 2005; Bielefeldt et al., 2002). Histological analyses further demonstrate changes in neuropeptide expression in nodose neurons of animal with gastric ulcers (Zalecki et al., 2020).

Clinical manifestations of these diseases—including anorexia, weight loss, abdominal pain, nausea, and high risk of infections—are consistent with vagal dysfunction (Davis and Kellerman, 2022; Lindgren et al., 1993; Ciesielczyk et al., 2017). Axonal damage and hypertrophic nerve fibers are consistently observed in gut samples from patients with inflammatory intestinal diseases (Lindgren et al., 1993; Mawe, 2015). While many of these axons likely belong to enteric neurons, the dense vagal innervation of the gut suggests involvement of vagal afferents.

Anti-inflammatory treatments often provide rapid symptom relief, whereas full remission may require months, suggesting that structural neural injury may contribute to disease persistence. Although the reversibility of vagal changes remains unclear and definitive tracing studies are lacking, the available evidence supports moderate confidence that vagal neuropathy occurs in at least a subset of patients with chronic inflammatory gastrointestinal disease.

Inflammatory airway diseases, including asthma and chronic obstructive pulmonary disease (COPD), also strongly implicate vagal involvement. Vagal afferents innervating the airways exhibit hyperresponsiveness in allergic inflammation, asthma, and COPD models (Bergren, 2001; Kuo and Lai, 2008; Riccio et al., 1996; Undem et al., 1993). Clinical studies support this notion, demonstrating reduced cough thresholds in COPD (Doherty et al., 2000). Disease-associated changes in neurochemical phenotype and excitability have been reported (Dinh et al., 2005; Fischer et al., 1996; Myers et al., 2002; Zhang et al., 2008), and transcriptional profiling has revealed injury-associated gene expression programs in airway-innervating vagal afferents (Crosson et al., 2024), including upregulation of Npy1r. However, in allergic inflammation, asthma, and COPD, we are not aware of morphological or biochemical data that clearly distinguish neuropathy from plasticity (see also Box 1).

Immune–neural interactions likely contribute to this dysfunction. Allergic airway inflammation induces migration of immune cells into airway sensory ganglia (Kistemaker and Prakash, 2019), and enhanced parasympathetic tone inferred from the efficacy of anticholinergic therapies implicates altered vagal efferent signaling (Chen et al., 2025). However, overt structural nerve damage has not been convincingly demonstrated, and reversibility remains uncertain. Consequently, these changes are generally framed as neuroplasticity rather than neuropathy, although a neuropathic component cannot be excluded.

### 3.4 Physical Injury–Related neuropathy

Physical injury to the vagus nerve reliably induces molecular and cellular changes consistent with neuropathy. Surgical vagotomy, historically performed for peptic ulcer disease and widely used in experimental models, elicits profound transcriptional remodeling, altered excitability, axonal degeneration, and induction of vagal ATF3 (Ryu et al., 2010; Broberger et al., 2001; Merchant et al., 2026).

Unintentional vagal injury may be more common than generally appreciated. Many visceral surgical procedures inevitably damage vagal branches or terminals (Gautron et al., 2013). In mouse models of Roux-en-Y gastric bypass, dystrophic axons are observed at injury sites, ATF3 is induced in vagal sensory neurons, and vagal excitability is altered (Merchant et al., 2026). Given the global prevalence of bariatric surgery, a substantial number of patients may experience unrecognized vagal neuropathy, potentially contributing to postoperative symptoms such as nausea, dumping syndrome, and dysautonomia (Gautron, 2021).

Other visceral surgeries, including tumor resection, transplantation, and cholecystectomy, may similarly impair vagal integrity. For example, loss of the cough reflex has been reported following lung transplantation. Importantly, preservation of vagal integrity correlates with improved surgical outcomes (Yan et al., 2025).

### 3.5 Cancer and cancer therapies

Cancer may affect the vagus nerve directly through tumors involving vagally innervated organs, the nerve itself, or central vagal nuclei. Although rare, vagal schwannomas, neurofibromas, and paragangliomas are well documented (Sreevatsa and Srinivasarao, 2011; Luo et al., 2021; Thatal et al., 2025). Clinical manifestations commonly include hoarseness, cough, dysphagia, dyspnea, and autonomic disturbances. Beyond direct nerve involvement, malignancies arising in vagally innervated organs may also alter vagal structure and function. For example, vagal hyperexcitability is reported in a mouse model of liver cancer (Garrett et al., 2025). Moreover, elevated Atf3 expression has been reported in the mouse jugular and nodose ganglia of mice bearing melanoma lung metastases, a clear indication of neuropathy (Baruch et al., 2025). These findings suggest that tumors developing in visceral organs with dense vagal innervation, such as the lung, stomach, and pancreas, may secondarily impair vagal integrity and signaling.

Cancer therapies represent an additional risk. Chemotherapeutic agents such as cisplatin, paclitaxel, and vincristine induce peripheral neuropathy (Boehmerle et al., 2014), and vagal involvement has been inferred from clinical symptoms and imaging studies (Goron et al., 2019; Weissman-Fogel et al., 2008; Shultz et al., 2014). Radiation therapy has also been implicated, although evidence remains sparse (Hutchinson et al., 2008; Piani et al., 2020). Given the high burden of autonomic and gastrointestinal symptoms in cancer patients (Dantzer et al., 2012), vagal neuropathy remains an underexplored contributor.

### 3.6 Alcoholism

Peripheral neuropathy is a well-established complication of chronic alcohol use. Early clinical reports describe hoarseness, dysphagia, and baroreflex dysfunction suggestive of vagal involvement (Novak and Victor, 1974; Duncan et al., 1980). Postmortem histological analyses reveal fiber degeneration and myelin loss in the vagus nerve of individuals with alcoholism (Guo et al., 1987; Novak and Victor, 1974). These findings strongly support the presence of vagal neuropathy in alcoholism, likely driven by ethanol toxicity, nutritional deficiencies, and inflammatory mechanisms.

### 3.7 Aging, neurodegenerative, and autoimmune diseases

Normal aging has been associated with spontaneous degeneration of vagal efferent axons innervating rat cardiac ganglia (Al-Biltagi et al., 2022; Li et al., 2010) as well as degeneration of vagal afferent terminals in the gastrointestinal tract (Phillips et al., 2010). While these structural findings are compelling, it remains unclear to what extent they translate to humans. Specifically, we are not aware of any morphological studies examining the integrity of the vagus nerve in older versus younger human individuals.

Among neurodegenerative disorders, Parkinson's disease (PD) provides the strongest evidence for vagal neuropathy. α-Synuclein pathology is consistently detected in the vagus nerve (Braak et al., 2007; Nakamura et al., 2016), and histological studies demonstrate substantial loss of vagal motor neurons, with reports of up to 50% neuronal loss (Gai et al., 1992). Although gastrointestinal and autonomic symptoms are common in PD, their precise anatomical origin remains debated, as pathology may involve the vagus nerve, the enteric nervous system, the spinal cord, or a combination of these structures (Challis et al., 2020; Braak et al., 2006; VanderHorst et al., 2015).

Many autoimmune diseases are associated with painful peripheral neuropathy, including Sjögren's syndrome, systemic lupus erythematosus, multiple sclerosis, rheumatoid arthritis, and GBS. Among these conditions, evidence for vagal involvement is strongest in GBS, where ultrasonographic enlargement of the vagus nerve and prominent autonomic dysfunction are frequently observed (He and Lin, 2024; Liu et al., 2021). The density of myelinated fibers was approximately 15,000 per mm^2^, compared with 73,000 unmyelinated fibers per mm^2^ in healthy subjects (Shimizu et al., 2011). Strikingly, the density of myelinated fibers decreased to 1257 per mm^2^ in GBS. However, an important caveat is that this observation was based on a single individual. Experimental allergic neuritis models further support vagal involvement, demonstrating demyelination and altered neuropeptide expression within vagal fibers (Myers et al., 2002; Tuck et al., 1981; Kalimo et al., 1982).

Overall, aging is likely associated with spontaneous forms of vagal neuropathy and may predispose individuals to the development of vagal neuropathies in age-related diseases. While vagal neuropathy is well supported in GBS and PD, it remains poorly characterized in most autoimmune and neurodegenerative disorders, representing a significant gap in our current understanding.

### 3.8 Putative immune dysregulation associated with vagal neuropathy

The vagus nerve plays a key role in immune modulation through bidirectional interactions with inflammatory responses. Vagal afferent fibers are particularly sensitive to inflammatory cytokines and prostaglandins (Huerta et al., 2025; Ek et al., 1998). Pathogen-associated molecular patterns (PAMPs) can also directly stimulate vagal afferent fibers (Jia et al., 2021; Liu et al., 2025; Ruhl et al., 2020; Meseguer et al., 2014). Excessive or chronic inflammation, as seen in various disorders discussed in this article, could contribute to vagal neuropathy by directly affecting these neurons. If this hypothesis is confirmed, early administration of anti-inflammatory drugs during inflammatory insults or infections may partially prevent the development of vagal neuropathy—though this has yet to be demonstrated.

On the other hand, once vagal neuropathy has been established, it may lead to immune dysregulation, contributing to the pathophysiology of neuropathy that affects both vagal and non-vagal neurons. Vagal damage could potentially alter immune responses at local or systemic levels, indirectly contributing to disease symptoms and the progression of neuropathy. Studies on vagus nerve stimulation (VNS) have established that electrical stimulation of the vagus nerve generally suppresses the release of systemic pro-inflammatory cytokines (Somann et al., 2019; Komegae et al., 2018; Falvey et al., 2024). Conversely, vagotomy, which can be considered an experimental model of vagal neuropathy, is known to elevate systemic inflammation and worsen the symptoms of numerous inflammatory challenges and infections (Verzele et al., 2024; Schulte et al., 2014; Ghia et al., 2008). One theoretical consequence of vagal hypoactivity due to neuropathy is an increased risk of chronic low-grade inflammation and heightened susceptibility to inflammatory conditions. On the other hand, vagal hyperactivity could exert immunosuppressive effects, potentially modulating T cell function and cytokine production. However, literature remains limited, and it is difficult to predict the outcomes of vagal neuropathy on immune function with certainty.

In fact, the impact may depend on the type of neuropathy and the primary etiology. For instance, in bacterial lung infection, vagal afferents contribute to the suppression of inflammation in the lungs. Ablation of vagal TRPV1 neurons using the diphtheria toxin system improves neutrophil recruitment, reduces lung bacterial burden, and enhances survival rates (Baral et al., 2018). However, depending on the context, vagal signaling may play either a beneficial or deleterious role. For example, during influenza infection, vagal nerve ablation is associated with vagal neuropathy (as evidenced by ATF3 expression) and worsens survival outcomes (Almanzar et al., 2025). Thus, vagal neuropathy can have both protective and detrimental effects depending on the immune context. Further research is needed to fully characterize the immunological sequelae of vagal neuropathy and determine its precise role in immune modulation across different disease contexts.

## 4 Conclusion

While vagal neuropathy is clinically recognized in specific contexts (e.g., laryngeal neuropathy), its broader integrative roles have not been closely examined. Vagal neuropathy is likely to coexist with other forms of peripheral neuropathy, such as diabetes-related, aging, or autoimmune neuropathies. As a result, it may be an underrecognized contributor to a broad spectrum of physical, mental, and immune symptoms. Although substantial evidence documents vagal abnormalities, these are seldom framed within the context of neuropathy, reflecting a historical research focus on painful somatosensory neuropathies and limited diagnostic tools for the vagus nerve. Importantly, clinical vagal neuropathies reflect structural or functional impairment of the nerve, whereas many emerging studies examine vagal pathways as modulators of systemic physiology. Recognizing vagal neuropathy offers a unifying framework for understanding diverse symptoms across autonomic, inflammatory, metabolic, and neuropsychiatric domains.

This paucity of studies on vagal neuropathies is largely a result of the historical dominance of research on painful neuropathies, particularly those associated with dorsal root ganglion (DRG) neuron dysfunction, which has shaped both experimental and clinical approaches. While such neuropathies are highly noticeable due to their direct impact on cutaneous sensory function, the vagus nerve's role remains less appreciated, primarily because clinicians have limited tools to assess its function. Consequently, vagal neuropathy is often suspected but traditionally studied in isolated contexts. We argue that vagal neuropathies are not only varied and common but also strongly linked to significant health issues.

The implications of recognizing vagal neuropathy are far-reaching. First, many unexplained symptoms could be attributed, at least in part, to vagal dysfunction. Second, treatments for pain might influence the vagus nerve, given that nociceptive receptors are often expressed in vagal neurons (Kupari et al., 2019). Third, future therapeutic strategies may focus on modulating abnormal vagal signaling directly. Although this is challenging due to limited access to the vagus nerve and the gaps in our understanding of its molecular composition, progress is being made. Fourth, there is an urgent need for reliable biomarkers and diagnostic tools to assess vagal function, encompassing both afferent and efferent components, as existing methods are often unreliable. Perhaps new microelectrode systems currently under development may help diagnose vagal neuropathy in humans in the future (Ottaviani et al., 2020).

Looking ahead, the field should prioritize the development of tailored therapies for vagal neuropathy, focusing on neuronal survival, regeneration, excitability, and the diverse symptoms associated with vagal dysfunction.

## CRediT authorship contribution statement

**Bandy Chen:** Data curation, Validation, Writing – review & editing. **Laurent Gautron:** Conceptualization, Project administration, Visualization, Writing – original draft, Writing – review & editing.

## Declaration of competing interest

The authors have no conflicts to declare.

## Data Availability

No data was used for the research described in the article.
